# Combined effect of thermosonication and natural preservatives on the quality of sugarcane juice

**DOI:** 10.1016/j.fochx.2025.102861

**Published:** 2025-08-02

**Authors:** Tehmina Bibi, Atif Liaqat, Tariq Mehmood, Rabia Iqbal, Muhammad Nadeem, Ashiq Hussain, Farhan Saeed, Muhammad Afzaal, Suleiman A. Athawab, Robert Mugabi, Yash D. Jagdale, Gulzar Ahmad Nayik, Tawfiq Alsulami

**Affiliations:** a Institute of Food Science and Technology, Khwaja Fareed University of Engineering and Information Technology, Rahim Yar Khan-64200, Pakistan; b Department of Bioscience, COMSATS University, Islamabad, Sahiwal Campus, Pakistan; c Institute of Food Science and Nutrition, University of Sargodha, Punjab Food Authority, Lahore, Pakistan; d Department of Food Science, Government College University Faisalabad, Pakistan; e Department of Food Science & Nutrition, College of Food and Agricultural Sciences, King Saud University, Riyadh 11451, Saudi Arabia; f Department of Food Technology and Nutrition, Makerere University, Kampala, Uganda; g Department of Food Science and Nutrition, University of Minnesota, 1334 Eckles Avenue, St. Paul, MN 55108, United States of America; h Marwadi University Research Centre, Department of Microbiology, Marwadi University, Rajkot-360003, Gujarat, India

**Keywords:** Sugarcane juice, Thermosonication, Shelflife, Natural preservatives, Sensory evaluation

## Abstract

Sugarcane juice (SCJ) is highly perishable, requiring effective preservation to maintain quality. Traditional methods ensure microbial safety but compromise juice quality, prompting exploration of alternatives. This study evaluated the synergistic effects of thermosonication (TS) and natural preservatives (NP) on SCJ quality, including physicochemical, viscosity, phytochemical, microbial, and sensory attributes. SCJ samples were treated with NP (T1), TS (65 °C, 15 min) (T2), both NP and TS (T3), or left untreated (control) and stored at 4 °C for 28 days. T3 exhibited minimal changes in total soluble solids, significantly reduced polyphenol oxidase activity, and lowered microbial load. T3 and T1 increased total phenolic content and antioxidant activity due to NP, with T3 retaining these best during storage. T3 also maintained stable viscosity and superior sensory attributes, particularly color and appearance, outperforming other samples. Combining NP and TS offers an eco-friendly, effective alternative to traditional preservation, enhancing SCJ quality, safety, and shelf life.

## Highlights

• Thermosonication and natural preservatives improved sugarcane juice shelf life.
• Combined treatment retained TPC, antioxidant activity, and sensory quality.
• Microbial load and PPO activity significantly reduced in TS + NP treated samples.
• Viscosity and turbidity remained stable in TS + NP treated sugarcane juice.
• Combined TS and NP treatment proved superior to individual methods for SCJ safety.

## 1 Introduction

Fruit and vegetable juices have gained high international recognition for their nutritional and functional advantages. These juices are significant sources of vitamins, minerals, and bioactive constituents which demonstrate distinct health-beneficial attributes. (Hussain et al., 2024; Yıkmış et al., 2025). Now a day, there is a rising demand for natural functional beverages (Silva et al., 2025).

Sugarcane (*Saccharum officinarum*) is a perennial tree plant growing in warm and temperate regions worldwide, especially in tropical and subtropical climates. It plays an important role in global agriculture, serving as a primary source of sugar production and known as “noble cane” (Afghan et al., 2023; Younos & Embaby, 2024). Pakistan ranks 5th globally in sugarcane cultivation, making it one of the country's key cash crops. (FAOSTAT, 2024).

Sugarcane juice (SCJ) offers numerous benefits with good antioxidant activity and economic affordability. With refreshing and nutritious qualities, it stands out as a popular beverage worldwide. (Hussain et al., 2024; Khan et al., 2021). Phenolic profile of SCJ includes caffeic acid, vanillic acid, chlorogenic acid, and 4-hydroxybenzoic acid significantly which play a crucial role as antioxidants and make it highly effective in boosting immunity and fighting diseases like prostate and breast cancer (Rungjang et al., 2022; Y. Yang et al., 2022).

SCJ is extremely perishable due to high water activity and rich nutrient content. Fermentation starts in SCJ as the time passes. Also, it is highly susceptible to fungi, bacteria, and other microbes. Naturally present enzymes and other chemical reactions are the other concerning issues. These problems limit its utilization to only be consumed as fresh within a few hours of extraction. (Tarafdar & Kaur, 2022; Younos & Embaby, 2024). Conventional heat treatment significantly addresses the food safety concerns of SCJ, however, high temperature and prolonged treatment time negatively affect the quality, nutritional attributes, and sensory appeal by darkening color and burnt flavor, ultimately chemical additives being utilized which also pose health risks to consumers (Mishra et al., 2011; Panigrahi et al., 2023; Wang et al., 2022).

To overcome the drawbacks of conventional treatments, researchers are investigating alternative combined thermal and non-thermal technologies to increase the shelf life while preserving the quality attributes (Allai et al., 2023). In alternative technologies, sonication is a promising non-thermal technology for deactivating microorganisms and enzymes in liquid food products while retaining quality and safety (Safwa et al., 2024). Ultrasound treatment at 20 or 40 kHz effectively preserves juice by creating voids in liquid molecules through ultrasonic waves, resulting in high shearing force with localized temperature changes (Paniwnyk, 2017). While sonication alone may not sufficiently reduce microbial levels, it has been investigated in combination with moderate heat, which is known as Thermosonication (TS), to achieve effective microbial safety without compromising quality, and also enhance phenolic content (Yıkmış et al., 2025). These feature makes the TS a viable alternative technique (Bhutkar et al., 2024). TS has emerged as an effective alternative to traditional thermal processing methods, capable of preserving juices while minimizing the loss of nutrients and maintaining and enhancing quality at lower temperatures (Lepaus et al., 2023; Silva et al., 2025). Also, it is environmentally friendly producing low wastes (Abdulstar et al., 2023; Yıkmış et al., 2025).

Organic matter in SCJ hinders the inactivation of microorganisms, and the natural microflora in SCJ demonstrates higher resistance (Garud et al., 2017). To counter this problem, lowering pH with lemon and adding ginger as a natural antimicrobial can act as an additional measure along with TS against microbial regeneration. Adding lemon juice to SCJ, lowers its pH, destabilizes peroxidase (POD), and improves taste (Kunitake et al., 2014). It is rich in citric acid, vitamin C, flavonoids, and minerals, serves as a characteristic additive while upgrading wholesome esteem (Vujčić Bok, Šola, & Rusak, 2022). Ginger (*Zingiber officinale*) has long been esteemed in conventional medicines for its antimicrobial, anti-inflammatory, and antioxidant properties. It has been used as both a food ingredient and a medicinal herb, with roots dating back to old India and China. Scientific studies highlight its antimicrobial potential, especially in inhibiting pathogenic bacteria and fungi due to its bioactive compounds like gingerol and phenolics which play a key part in food preservation (Laelago Ersedo et al., 2023a).

Despite sugarcane juice (SCJ) being a highly nutritious and widely consumed fresh beverage, its rapid perishability has hindered large-scale commercialization. Existing preservation methods—primarily thermal or chemical—compromise sensory and nutritional quality, limiting consumer appeal. To date, limited research has explored the synergistic application of thermosonication and natural preservatives, particularly ginger and lemon, in enhancing SCJ's shelf life. This study is novel in its integration of a clean-label preservation strategy combining moderate thermal-ultrasound treatment with plant-derived bioactives, targeting both microbial safety and quality retention. The necessity arises from the industry's growing demand for eco-friendly, cost-effective, and minimally processed beverage preservation technologies. Thus, this research aims to bridge this gap by offering a scalable and sustainable solution for SCJ processing, with enhanced physicochemical, microbial, and sensory attributes suitable for commercial application.

## 2 Materials and methods

### 2.1 Procurement of raw material

Matured, fresh, healthy, and disease-free stems of sugarcane rind of variety (CP72–2086) were harvested from the local farm of the village of Sheikh Abdul Sattar, Khanpur, Rahim Yar Khan, South Punjab, Pakistan. The high-quality sugarcane was taken to a laboratory for further processing. The ginger, lemon, and salt were brought from the local market.

### 2.2 Sample preparation

#### 2.2.1 Sugarcane juice extraction

Sugarcane rind was graded and sorted based on its color and appearance quality. Then it was thoroughly cleaned, washed with tap water to remove dust and soil, and peeled manually with a stainless-steel knife, removing the buds from the rind. SCJ is extracted through an electric power-operated stainless steel extractor machine (Local Model of Pakistan: YF-L80, NO.202211, Power: 1100 W). After extraction, filtration was done with a 1 mm mesh sieve and six layers of muslin cloth to remove debris and insoluble matter.

#### 2.2.2 Natural preservatives preparation

Firstly, ginger and lemon were graded and sorted, and washed with tap water. Two beakers were sterilized with hot water. Ginger was peeled, cut into pieces, and crushed into a paste, from which juice was extracted and filtered using a muslin cloth. Lemons were cut, manually squeezed, and their juice was similarly filtered using a muslin cloth to remove seeds and impurities. (Khare et al., 2012).

### 2.3 Experimental design

Freshly obtained SCJ was then taken for further treatment, in which the SCJ was partitioned into four equal batches: T0, T1, T2, and T3, as given in Table 1. The first batch (T0) served as a control without treatment. The 2nd batch (T1) received natural preservatives, consisting of 0.6 mL ginger, 3 mL lemon juice, and 1 mg salt per 100 mL sugarcane juice, following optimized preservative concentrations from Rajendran et al. (2017). The third batch (T2) was treated with thermosonication using an ultrasonic bath operating at a frequency of 37 kHz and 70 % power output (500 W) (Elmasonic Easy 30H, Model: YF-L80, Germany) at 65 °C for 15 min (Abid et al., 2014). The 4th batch (T3) combined NP and TS treatment, incorporating firstly 3 mL lemon juice, 0.6 mL ginger, and 1 mg salt per 100 mL sugarcane juice, followed by ultrasound treatment at 65 °C and 37 kHz for 15 min.

**Table 1:** Treatment plan for sugarcane juice.

Treatments	Samples	Natural preservatives	Thermosonication
T_0_	Control	**_**	**_**
T_1_	Sugarcane juice	(3.0 mL lemon juice+1 g salt and 0.6 mL ginger per 100 ml SCJ)	**_**
T_2_	Sugarcane juice	**_**	(37 kHz, 65 °C for 15 min)
T_3_	Sugarcane juice	(3.0 mL lemon juice+1 g salt and 0.6 mL ginger per 100 mLSCJ)	(37 kHz, 65 °C for 15 min)

### 2.4 Storage and analysis

All treated and control samples were stored at refrigeration temperatures (4 °C) for 28 days to mimic real-world conditions, and routine analyses were conducted weekly over a month to assess the samples. The physico-chemical analysis, rheological analysis, phytochemical analysis, microbial analysis, and sensory evaluations were conducted.

### 2.5 Physicochemical analysis

Throughout the storage period, samples were regularly analyzed for changes in the physicochemical parameters, including pH, TSS, titrable acidity, reducing sugar, polyphenol oxidase activity, and turbidity. These measurements were conducted using standard methods.

#### 2.5.1 Total soluble solids (TSS)

TSS denotes the concentration of soluble constituents, often measured in degrees Brix, indicating sweetness or concentration. TSS measurement relies on refractive index changes using a refractometer. Following the method by Ali et al. (2023) SC juice TSS was determined using a refractometer with a high-refractive-index prism. The procedure began with prism cleansing using distilled water and tissue paper for zero adjustment. A drop of sugarcane juice at 25 °C was placed on the prism, covered, and positioned toward the light for a refractometer reading. The reading was noted where the white and dark colors met as a thin line. For subsequent samples, the prism was cleaned and adjusted before repeating the process.

#### 2.5.2 pH

pH of SCJ samples was assessed following the method outlined by Liao et al. (2018). Initially, the digital pH meter (Model 361, Systronic) was calibrated with buffer solutions with pH 4 and 7. Subsequently, for each new sample, the pH meter was calibrated, ensuring the knob was rinsed using distilled water. The pH of the sample was measured in a beaker at room temperature. Once the pH meter had stabilized, the recorded pH value was noted. Following this, the pH meter knob was rinsed again with distilled water before being immersed in another sample.

#### 2.5.3 Titrable acidity (TA)

Titratable acidity of the SCJ was determined following the method of Zaman et al. (2023). Firstly, 10 mL of the sample was measured into a graduated beaker, followed by the addition of 250 mL of distilled water. Next, 2–4 drops of phenolphthalein dye were introduced into the solution. Sodium hydroxide (NaOH) with 0.1 N of solution, prepared by dissolving 4 g of NaOH in 1  L of distilled water, was then titrated against the acid present in the SCJ sample. The NaOH volume was recorded before and after titration, noting the appearance of a pink color. Both the volume of alkali before and after titration were recorded. Since citric acid was the predominant acid in the sample, its equivalent weight was used as the calculation factor, expressed as a percentage.

*Titratable acidity % = Volume of NaOH used in mL × 0.009 / volume of sample in mL× 100.*

#### 2.5.4 Reducing sugar (RS)

The method described by Dalai and Panda (2021) was followed to determine the reducing sugar content. DNSA (dinitro salicylic acid) reagent was prepared by dissolving 50 mg of sodium sulphite and 200 mg of crystal phenol in 1 % sodium hydroxide solution. Making the overall volume up to 100 mL the reagent was stored at 4 °C. Sodium sulphite was added in the mixture at the time of use, as the reagent deteriorates during long time storage. To prepare the Rochelle salt solution, 40 g of potassium sodium tartrate was dissolved in distilled water, making the total volume 100 mL. To measure the reducing sugars, SCJ sample (0.1 mL) was mixed with 5.9 mL of distilled water in a test tube. Subsequently, 3 mL DNSA reagent was introduced into the mixture, followed by heating the test tube in boiling water for 5 min. Post-heating, 1 mL of Rochelle salt solution was gently incorporated into the warm mixture to prevent dissolving oxygen. After allowing the solution to cool, absorbance in the UV–VIS spectrophotometer at 510 nm was measured and compared with the standard curve

#### 2.5.5 Polyphenol oxidase activity (PPO)

To assess the PPO activity, the methodologies adapted are outlined by Geremias-Andrade et al. (2020). 1 mL of diluted sample in deionized water (1:10) was prepared and 7 mL of 0.2 M phosphate buffer solution. (pH 5.5) was added in a test tube. Temperature of the sample was maintained through a heat bath at 35 °C for 10 min, then 0.5 mL of 0.1 % hydrogen peroxide (H_2_O_2_) with 1.5 mL of 0.05 % guaiacol was added in it. The concoction was subjected to magnetic stirring for 15 s before being immersed in a heat bath set at 35 °C for 15 min. A blank sample was concurrently prepared by diluting the juice in deionized water. Absorbance of the samples was measured at 425 nm using a UV–VIS spectrophotometer (Labomed USA). Enzyme activity was quantified in units per milliliter (U/mL), with one unit equating to the amount of enzymatic extract capable of increasing absorbance at 425 nm for PPO, respectively, at rates of 0.001 units per min. (Kunitake et al., 2014).

*POD Activity Unit of ppo/mL = (Ab*_*sample*_*– Ab*_*blank*_*) / 0.001× time.*where, Ab _sample_ was the absorbance values for a sample, and the Ab _blank_ was the blank, respectively; t denoted the reaction time.

#### 2.5.6 Turbidity

Turbidity measurements reflect the number of suspended particles in the juice, indicating its clarity. Turbidity was measured using a turbidimeter, with results expressed in Nephelometric Turbidity Units (NTU). The method as described by Demir and Kılınç (2019) was followed. The meter was first checked for its accuracy using one or more turbidity standards. The well mixed SCJ sample was then placed in the cuvette, priorly cleaned with turbidity free water, for the analysis. Reading appearing on the device was noted.

### 2.6 Viscosity

Viscosity readings help determine the juice's flow behavior. The viscosity of the sample was measured using a rotary viscometer, with results recorded in centipoise (cP). A 10 mL sample of SCJ was placed into a sterilized container at a temperature of 25 °C, and the viscometer was set to operate at 100 rpm. The measurement was performed in triplicate to ensure accuracy and consistency. This method followed the procedure outlined by Oladunjoye and Awani-Aguma (2023).

### 2.7 Phytochemical analysis

#### 2.7.1 Total phenolic content

To determine the total phenolic content, we followed the methodology outlined by Ali et al. (2023). Initially, 1 mL of the juice sample was combined with a 1 mL aliquot of 0.10 mg/mL gallic acid solution in methanol in a beaker. This mixture was then blended with 5 mL of Follin-Ciocalteu reagent, diluted tenfold with distilled water in the cylindrical flask, and supplemented with 4 mL of Sodium carbonate (Na_2_CO_3_) (20 %). After allowing the reaction to proceed for 45 min. at room temperature, the prepared sample was placed in a small bucket fitted with a UV Spectrophotometer. Then, absorbance readings were taken at 765 nm using resonance. This process was repeated in triplicate to ensure accuracy and reliability. Total phenolic content was calculated as gallic acid equivalents (GAE) in mg/mL.

#### 2.7.2 Antioxidant activity (AA)

A stock solution of 2,2-diphenyl-1-picrylhydrazyl (DPPH) was prepared with methanol, in which 0.025 mg DPPH in powder form was added to 100 mL of methanol, mixed thoroughly, covered with aluminum foil, and then incubated at room temperature in a dark place for 30 min. For the DPPH assay, 100 μl of filtered samples with 3 mL of methanolic DPPH solution, and a blank sample was prepared by adding 3 mL of methanolic DPPH solution and 100 μl of methanol in the test tube, and each test tube was covered with aluminum foil and incubated in the dark at room temperature for 30 min. Absorbance was measured at 517 nm using a UV-VIS Spectrophotometer, and the DPPH free radical-scavenging activity was calculated using the following equation.

*Antioxidant activity (DPPH %) =* *Absorbance of control sample* *− Absorbance of treated sample/ Absorbance of control sample × 100.*

### 2.8 Microbial analysis

The microbial analysis followed Adulvitayakorn et al. (2020) with slight changes, assessing total plate count (TPC) and yeast and mold count (YMC) tests were conducted. Plate count agar (PCA) from Sigma-Aldrich, USA, served as the growth medium for TPC using the pour-plate method. Potato dextrose agar (PDA) from bio PLUS Chemicals) served as the growth medium for YMC analysis, using the spread plate method. Growth media were prepared according to standard instructions: 2.4 g of PCA in 100 mL of distilled water, heating in a water bath until clear, and covered with aluminum foil; 3.9 g PDA in 100 mL of distilled water. Test tubes, both nutrient media, and diluents (peptone water) were then put in an autoclave machine at 121 °C for 15 min. and then immediately placed in a sterilized laminar airflow safety cabinet, which was sterilized with UV light. Each test tube was filled with 9 mL of peptone water and covered with the help of aluminum foil. Dilutions (10^−1^ to 10^−4^) were made by transferring 1 mL of the sample into test tubes. A 0.1 mL aliquot of diluted samples was plated: for TPC, 20 mL plate count agar was added and solidified before incubation at 37 °C for 20–24 h; for YMC, 20 mL PDA was spread, forming a gel, and 0.1 mL of the 10^−4^ dilution was pipetted into YMC labeled Petri dishes using a micropipette (Gilson Pipetman, 060087 N). Incubation for YMC lasted minimum of 72 h at 25 °C. Colony counts were recorded, with results expressed in log colony-forming units per mL (log CFU/mL).

*CFU/mL = Number of colonies x Dilution factor / Volume of sample plated.*

Colony counts were converted into log CFU/mL by calculating the colonies in Log ^10^.

*Log CFU/mL = Log* ^*10*^ *(Number of colonies).*

### 2.9 Sensory evaluation

A sensory evaluation was conducted on SCJ samples following the approach outlined by Dhansu et al. (2023). Ten semi-trained assessors participated in the evaluation, rating the juices based on color, odor, taste, appearance, and overall acceptability. For sensory evaluation, panelists were initially asked to take two quick sniffs of marked samples, and they were instructed to evaluate each attribute of samples using a 9-point hedonic scale, where point from 1 = dislike extremely, to 9 = like extremely, as detailed in the sensory evaluation score card. They were also advised to rinse and swallow water before the analyses of every sample to cleanse their palate. The scores above 5 indicated acceptance of the juice.

### 2.10 Statistical analysis

The results were reported as the means of triplicate samples along with their standard deviations (± S.D.). Statistical significance was determined using a two-way ANOVA followed by Tukey's test, with data analysis performed using SPSS 8.1 (SPSS, Chicago, USA).

## 3 Results and discussion

The primary goal is to gain a comprehensive understanding of the effects of innovative techniques of thermosonication and natural preservatives in a combined form on the SCJ quality parameters, such as physicochemical properties, microbial load, phytochemical properties, and sensory properties during 28 days of storage at 4 °C.

### 3.1 Physicochemical analysis

Physicochemical parameters such as pH, total soluble solids, titratable acidity, reducing sugar, and turbidity are crucial indicators of deterioration in SCJ quality. In the storage analysis of the control sample, usually, TSS and pH levels decrease, and TA, reducing sugar, and PPO activity increase. These changes likely occurred due to microbial activity, such as bacteria, yeast, and molds in the juice contributing to fermentation by utilizing sugars to produce acids, leading to a decrease in TSS and pH and increases in TA and reducing sugar, as also noted in their study. (Dhansu et al., 2023). Various treatments and storage durations influenced the statistical data regarding these Physico-chemical parameters in SCJ.

After treatment, slight pH, TA, and TSS variations were observed in T1 and T3 samples due to the addition of natural preservatives (ginger and lemon juice). However, the T2 sample does not significantly alter pH, TA, or TSS and significantly lowers the PPO activity. During storage of 28 days and at 4C° in all of these samples, the T3 and T2 samples showed maximum retained pH, TSS, TA, and very slight increases in PPO activity and reducing sugar, as reported in Table 2.

**Table 2:** Effect of natural preservatives, thermosonication, and combined treatment of TS and NP, and storage time on the total soluble solids (TSS) and pH of sugarcane juice.

Physiochemical properties	Treatments	Storage Days				
		0	7	14	21	28
TSS (°B)	T0	12.50 ± 0.03^bc^	12.33 ± 0.04^ij^	12.19 ± 0.02^m^	12.01 ± 0.03^n^	11.80 ± 0.02^o^
	T1	12.59 ± 0.02^a^	12.53 ± 0.03^b^	12.43 ± 0.01^ef^	12.29 ± 0.01^k^	12.15 ± 0.01^m^
	T2	12.41 ± 0.01^fg^	12.38 ± 0.02^gh^	12.35 ± 0.02^hi^	12.30 ± 0.03^jk^	12.24 ± 0.01^l^
	T3	12.52 ± 0.02^b^	12.51 ± 0.01^bc^	12.48 ± 0.03^cd^	12.46 ± 0.01^de^	12.43 ± 0.03^ef^
pH	T0	5.21 ± 0.02^b^	5.14 ± 0.03^c^	5.03 ± 0.03^g^	4.88 ± 0.02^j^	4.72 ± 0.02^m^
	T1	4.89 ± 0.03^ij^	4.85 ± 0.01^k^	4.78 ± 0.02^l^	4.64 ± 0.03^n^	4.49 ± 0.01^o^
	T2	5.24 ± 0.02^a^	5.21 ± 0.01^b^	5.16 ± 0.01^c^	5.11 ± 0.06^d^	5.04 ± 0.03^fg^
	T3	5.09 ± 0.01^de^	5.06 ± 0.02^ef^	5.02 ± 0.04^g^	4.96 ± 0.01^h^	4.91 ± 0.01^i^

Different letters in a column denote significant results (*p* ≤ 0.05), whereas the same letters denote non-significant results. TS = Thermosonication; NP = Natural Preservatives.

T0 = Control sample without natural preservatives and thermosonication.

T1 = Non thermosonicated Sugar cane juice with natural preservatives.

T2 = Thermosonocated sugar cane juice with no natural preservatives.

T3 = Thermosonicated Sugar cane juice with natural preservatives.

#### 3.1.1 TSS

TSS in SCJ encompasses all dissolved solids, including sugars and various compounds. Each gram of sucrose dissolved in 100 mL of solution is equivalent to one degree Brix. The initial TSS of the T0 sample yielded a value of 12.50 in Table 2. In T1, the addition of natural preservatives results in a slight effect on TSS. Throughout the storage period, TSS of T1 exhibited significant fluctuations but less than the T0 sample. Due to its high sugar content, SCJ is highly susceptible to rapid spoilage, primarily caused by bacteria, yeast, and mold that metabolize sucrose into organic acids and ethanol, with enzymes facilitating sucrose inversion. But the slight preservation effect is attributed to the antimicrobial properties of ginger and the pH-lowering effect of lemon. However, the TSS decline may be attributed to the absence of heat treatment in T1 samples, as indicated by Khare et al. (2012), who suggested that heat treatment in conjunction with natural preservatives to efficiently kill microbes could effectively prolong the preservation period of SCJ.

The TSS values of T2 juices exhibited minimal reduction, as reported in Table 2. The findings suggest that the application of TS processing did not induce significant changes in the TSS values of sugarcane juice, which is consistent with previous studies by Nayak et al. (2020). The addition of ginger and lemon with the TS treatment of SCJ has slightly change the TSS. But during storage at refrigeration temperatures, in T3 samples, a non-significant effect was observed on TSS. The T3 and then T2 sample has effectively retained the TSS of SCJ.

#### 3.1.2 pH

pH is calculated through the negative logarithm of hydrogen ion concentration. On the pH scale, ranging from 0 to 14, neutrality is represented by 7, with values below indicating acidity and those above indicating alkalinity. In the context of SCJ, pH acts as an indicator of spoilage within control samples. (Kanagaraj et al., 2024). In T1, a notable decrease in pH from 5.21 to 4.89 was observed immediately after the addition of natural preservatives to the SCJ, as reported in Table 2. Similar pH-lowering effects following the addition of lemon and ginger have been documented previously. (Khare et al., 2012). While less significant changes in pH were occurs from day 0 to day 14, significant differences emerged onward day 14 to 28, potentially attributed to enzymatic and microbial activities. The microbial metabolism of carbohydrates during fermentation leads to the generation of acids, consequently lowering the pH. Similar pH reductions during a 28-day storage period at 4 °C have been documented in pineapple juice. (Mala et al., 2021).

Conversely, in T2, TS treatment did not induce significant alterations in the pH after treatment; a slight decrease in pH occurred throughout the storage period. This observation aligns with findings reported by Bhutkar et al. (2024), who applied the TS treatment to pineapple juice. In T3, the inclusion of NP along with TS treatment led to a slight reduction in pH, attributed to the addition of lemon, which contains citric acid. This combined treatment exhibited a notably high inhibition effect on microbial load, consequently resulting in a lack of significant pH alterations in the T3 SCJ sample during storage among all samples, as reported in Table 2. A study by Liao et al. (2018) investigated the combined effects of thermosonication and 100 ppm nisin on fresh apple juice and found that the treatment retained 89 % of ascorbic acid and pH and TSS values, thereby extending shelf life to 15 days at 8 °C.

#### 3.1.3 Titratable acidity

TA is a critical parameter. The shelf life of juice is intricately linked to its TA, with higher acidity often leading to reduced shelf life due to various biochemical processes such as hydrolysis, fermentation, and oxidation. The initial TA value of the control sample was measured at 0.24, as written in Table 3, which increased significantly during the storage period. This increment is ascribed to the production of acetic and lactic acids, facilitated by the heightened microbial load in the T0.

**Table 3:** Effect of natural preservatives, thermosonication, combined treatment of TS and NP, and storage time on the titrable acidity (TA) and reducing sugars (RS) of sugarcane juice.

Physiochemical properties	Treatments	Storage Days				
		0	7	14	21	28
TA (%)	T0	0.24 ± 0.01^k^	0.29 ± 0.02^hi^	0.33 ± 0.01^fg^	0.39 ± 0.01^cd^	0.43 ± 0.03^b^
	T1	0.31 ± 0.02^gh^	0.33 ± 0.02^fg^	0.37 ± 0.02^de^	0.40 ± 0.01^c^	0.46 ± 0.01^a^
	T2	0.26 ± 0.01^jk^	0.27 ± 0.02^ij^	0.29 ± 0.01^hi^	0.31 ± 0.03^gh^	0.35 ± 0.02^ef^
	T3	0.32 ± 0.03^g^	0.33 ± 0.02^fg^	0.35 ± 0.03^ef^	0.35 ± 0.01^ef^	0.37 ± 0.01^de^
RS (%)	T0	0.84 ± 0.03^gh^	0.89 ± 0.01^fg^	0.95 ± 0.01^de^	1.14 ± 0.02^b^	1.27 ± 0.04^a^
	T1	0.85 ± 0.02^gh^	0.88 ± 0.03^gh^	0.92 ± 0.02^ef^	0.97 ± 0.0^d^	1.06 ± 0.02^c^
	T2	0.80 ± 0.03^k^	0.82 ± 0.02^jk^	0.85 ± 0.03^gh^	0.89 ± 0.04^fg^	0.93 ± 0.01^de^
	T3	0.82 ± 0.01^jk^	0.83 ± 0.01^ij^	0.85 ± 0.02^gh^	0.87 ± 0.02^gh^	0.89 ± 0.02^fg^

Different letters in a column denote significant results (p ≤ 0.05), whereas the same letters denote non-significant results. TS = Thermosonication; NP = Natural Preservatives.

T0 = Control sample without natural preservatives and thermosonication.

T1 = Non thermosonicated Sugar cane juice with natural preservatives.

T2 = Thermosonocated sugar cane juice with no natural preservatives.

T3 = Thermosonicated Sugar cane juice with natural preservatives.

TA slightly increased in T1 after the addition of NP, which remained stable for 2 weeks, as given in Table 2. This increase in TA may be due to the presence of citrus and ascorbic acid in lemon and ginger juice, which increases the effect of TA after the addition of ascorbic acid, also noted by the study of (Bhukya et al., 2023). But in T2, the TA values of thermosonicated samples did not exhibit significant variation after treatment, as given in Table 3, consistent with findings reported by Fatima et al. (2023), who utilized microwave heating-TS for a blend of muskmelon and sugarcane juice. And slight increases during storage, consistent with findings reported by Nayak et al. (2020).

In T3, the combined effect of NP and TS resulted in much non-significant increase in TA values, with maximum stability observed during storage as reported in Table 3, and this effect is consistent with the combined treatment of TS with natural antimicrobial. (Zhao et al., 2021). The synergy between NP, which increased TA values, and TS, known for its stability-inducing effects, contributed to a significant reduction in enzymes and microbial load, thereby maintaining stable TA values throughout storage.

#### 3.1.4 Reducing sugar

In the T1 sample, after the addition of NP, there was no significant effect on the reducing sugar content as reported in Table 3, a finding also noted by Khare et al. (2012). But RS increases during the storage period. Over time, the hydrolysis of sucrose in SCJ produces reducing sugars like fructose and glucose. (Febrianto Mulyadi et al., 2022). This increase in RS alters the delicate flavor of the juice, leading to an overly sweet and undesirable taste. (Tarafdar & Kaur, 2022). Microbial activity increases, reducing sugar content and deteriorating carbohydrates. In T2, TS treatment slightly changes the reducing sugar, as reported in Table 3. (Khan et al., 2021) found that US treatment at 30 °C for 40 min. Slightly increased RS, likely due to polysaccharide hydrolysis during treatment. However, Adulvitayakorn et al. (2020) observed that US treatment at 60 °C and 80 °C for 30 min. Slightly decreased reducing sugars, while conventional thermal processing caused a significant decrease. This suggests that US treatment alone at lower temperatures and longer durations slightly increases reducing sugar, whereas combining heat with sonication in short durations tends to reduce it. The reducing effect of the TS treatment here could be due to the slightly higher temperatures applied in a short time.

The combined treatment T3 results in a non-significant effect on RS. In their (Febrianto Mulyadi et al., 2022) Study, the combined treatment of Pulsed electrical field and ginger extract has been reported to have a slightly decreasing effect on RS. In the T3 sample, very less increases in RS during the 28-day storage period were shown as compared to T2 and T1, as reported in Table 3. When RS levels stay low during storage, it's a sign that the process (microbial activity) responsible for sugar breakdown is slowed down. According to (Beristain-Bauza et al., 2019) Antimicrobial compounds present in ginger may inhibit microbial growth, thereby reducing microbial activity and subsequently resulting in fewer increases in RS during storage.

#### 3.1.5 Polyphenol oxidase activity

In SCJ, PPO activity is pivotal in determining juice quality, as it catalyzes the enzymatic browning, which causes an undesirable reddish-brown color in SCJ and requires immediate inactivation to prevent browning. (Chauhan et al., 2017). The control sample, devoid of any treatment, exhibits rapid deterioration and browning post-extraction, with enzymatic activity markedly increasing during the 28-day storage period. This was also noted in the study by Geremias-Andrade et al. (2020). In T1, the addition of lemon, lowering the pH, results in decreased enzymatic activity to some extent, as given in Table 4, as PPO functions optimally at pH 7.2, thereby aiding in retaining juice quality during storage. (Kanagaraj et al., 2024). This effect has also been noted by Khan et al. (2021).

**Table 4:** Effect of natural preservatives, thermosonication, and combined treatment of TS and NP, and storage time on the polyphenol oxidase activity (PPO) and turbidity of sugarcane juice.

Physiochemical properties	Treatments	Storage Days				
		0	7	14	21	28
PPO (U/mL)	T0	4.92 ± 0.01^c^	5.13 ± 0.03^c^	5.36 ± 0.02^b^	5.50 ± 0.02^ab^	5.62 ± 0.02^a^
	T1	2.65 ± 0.03^f^	2.76 ± 0.01^ef^	2.89 ± 0.01^e^	3.11 ± 0.01^g^	3.36 ± 0.03^d^
	T2	1.50 ± 0.03^ij^	1.54 ± 0.02^hi^	1.59 ± 0.01^hi^	1.65 ± 0.01^hi^	1.72 ± 0.02^h^
	T3	1.03 ± 0.02^l^	1.07 ± 0.02^l^	1.13 ± 0.01^kl^	1.20 ± 0.01^kl^	1.29 ± 0.02^jk^
Turbidity (NTU)	T0	157 ± 0.3^cd^	153 ± 0.4^de^	148 ± 0.2^fg^	130 ± 0.3^k^	121 ± 0.3^l^
	T1	166 ± 0.4^a^	162 ± 0.1^ab^	157 ± 0.1^cd^	141 ± 0.1^i^	133 ± 0.1^jk^
	T2	151 ± 0.3^ef^	148 ± 0.1^fg^	146 ± 0.1^gh^	142 ± 0.4^hi^	137 ± 0.4^ij^
	T3	162 ± 0.2^ab^	161 ± 0.5^ab^	158 ± 0.4^bc^	155 ± 0.2^de^	151 ± 0.1^ef^

Different letters in a column denote significant results (p ≤ 0.05), whereas the same letters denote non-significant results. TS = Thermosonication; NP = Natural Preservatives; NTU = Nephelometric Turbidity Units.

T0 = Control sample without natural preservatives and thermosonication.

T1 = Non thermosonicated Sugar cane juice with natural preservatives.

T2 = Thermosonocated sugar cane juice with no natural preservatives.

T3 = Thermosonicated Sugar cane juice with natural preservatives.

T2 treatment showcases a significant reduction in PPO activity than T1, as reported in Table 4. This finding is also supported by Zhao et al. (2021), who demonstrated higher PPO inactivation rates with TS compared to conventional thermal treatment. This underscores the potential of TS as a technology for enzymatic inactivation. TS induces stable cavitation bubbles that collapse under extreme pressure and temperature, generating shock waves and intense shear in the liquid, resulting in protein structure alterations and enzyme inactivation. (Abdulstar et al., 2023) In a short time, the sample T3 exhibits a highly significant reduction in PPO activity. As TS effectively inactivates the PPO enzymes at low pH and the remaining few enzymes are unable to work optimally, a very slight increase in PPO activity has been observed in the T3 sample during storage as compared to the T2 and T1 samples.

#### 3.1.6 Turbidity

Turbidity is a key indicator of the stability and quality of juice, with higher turbidity indicating a more stable quality of juice. (H. Wang et al., 2022). Changes in turbidity can signal microbial growth or chemical changes, which are critical for maintaining product safety. (Karmakar et al., 2011). In this study, the control sample initially had high turbidity, which decreased over time, leading to sedimentation by the 28th day. In the T1 sample, turbidity increased after adding NP but decreased during storage, with suspended coarse particles observed. It may be due to the aggregation of fibers, proteins, and carbohydrates. In the T2 sample, after TS treatment at 65 °C for 15 min, turbidity was not much affected, as reported in Table 4. A similar study (Demir & Kılınç, 2019) Showed no significant effect on the turbidity of pumpkin juice at TS-60 °C compared to other treatments. However, turbidity in the T2 sample remained more stable during storage than in the T1 sample.

The turbidity of the T3 sample was slightly increased and show a more improved turbidity as compared to other treatment, after the combined treatment of NP and TS, and it exhibited the most stable turbidity, remaining uniform without aggregation and sedimentation during storage, unlike other samples, as reported in Table 4. This improvement may be due to TS, which breaks down SCJ and NP (lemon and ginger juice) components into smaller particles, ensuring uniform distribution. (Demir & Kılınç, 2019) Found that low-power ultrasonic treatment reduces pectin and breaks down large macromolecules, improving the turbidity of pumpkin juice. (H. Wang et al., 2022) Observed that US-UV treatment produced smaller particles. This enhances the stability of juice during storage by making particles easier to disperse and reducing interfacial tension, minimizing the gravitational effects of larger particles. The combined treatment of TS and NP likely stabilizes the SCJ by preventing particle aggregation and sedimentation, thereby maintaining higher turbidity. Additionally, factors such as pH stabilization, antimicrobial effects, and inhibition of enzymatic activity contribute to preserving the juice's rheological and physical properties and extending its shelf life.

### 3.2 Rheological analysis

#### 3.2.1 Viscosity

Viscosity, often called fluid thickness, helps to determine the SC juice's flow behavior, particularly during storage, as it helps to ensure product stability and consumer safety. The viscosity of the T0 sample was 3.71 cP, which was increased during storage as reported in Table 5, likely due to higher dextran content from polysaccharide-producing bacteria like Leuconostoc spp. that utilize stored sucrose to produce dextran via dextransucrase. (Singh et al., 2016)This leads to increased viscosity and a slimy texture, which negatively affects juice quality.

**Table 5:** Effect of natural preservatives, thermosonication, and combined treatment of TS and NP and storage time on the viscosity of sugarcane juice.

Rheological properties	Treatments	Storage Days				
		0	7	14	21	28
Viscosity (cP)	T0	3.71 ± 0.02^fg^	3.78 ± 0.02^e^	3.87 ± 0.02^d^	4.08 ± 0.01^b^	4.21 ± 0.02^a^
	T1	3.69 ± 0.03^g^	3.73 ± 0.02^f^	3.79 ± 0.04^e^	3.86 ± 0.03^d^	3.95 ± 0.01^c^
	T2	3.52 ± 0.01^jk^	3.55 ± 0.03^j^	3.61 ± 0.02^i^	3.66 ± 0.04^h^	3.72 ± 0.01^f^
	T3	3.48 ± 0.02^l^	3.51 ± 0.01^kl^	3.55 ± 0.02^j^	3.59 ± 0.02^i^	3.65 ± 0.03^h^

Different letters in a column denote significant results (p ≤ 0.05), whereas the same letters denote non-significant results. TS = Thermosonication; NP = Natural Preservatives.

T0 = Control sample without natural preservatives and thermosonication.

T1 = Non thermosonicated Sugar cane juice with natural preservatives.

T2 = Thermosonocated sugar cane juice with no natural preservatives.

T3 = Thermosonicated Sugar cane juice with natural preservatives.

In the T1 sample, viscosity was not significantly changed after NP addition, but it started increasing during the two weeks of storage. Further, it increased, as reported in Table 5, possibly due to microbial activity or chemical changes. (Karmakar et al., 2011). (Febrianto Mulyadi et al., 2022), Also noted no significant effect on the viscosity of SCJ was noted when ginger extract was added to SCJ. The T2 sample, treated with TS, showed a significant decrease in viscosity. This reduction is likely due to cavitation, which breaks down larger molecules and homogenizes the juice, reducing viscosity due to lower intermolecular forces in SCJ. (J. Wang et al., 2020) and (Bhukya et al., 2023). Also, mild temperatures cause a decrease in viscosity.

In the T3 sample, viscosity decreases after treatment and but it is highly stable throughout the storage period, unlike the other samples, as reported in Table 5. For instance, Oner (2020) Found that adding nisin to green juice with thermosonication slightly increased viscosity. But, in the (Zhao et al., 2021) Study, a significant reduction in the viscosity of orange juice was found when nisin was combined with TS. Similarly, in my study, the combined treatment brought it decrease viscosity of the juice. But these variations depend on factors like solids content and particle size. (Oner, 2020), As well as treatment and temperature. By giving appropriate treatment that reduces microbial and enzymatic activity, the viscosity of juice can remain stable during storage.

### 3.3 Phytochemical analysis

The Phytochemical analysis of SCJ indicated notable findings regarding its total phenolic content and antioxidant activity, as shown in Table 6.

**Table 6:** Effect of natural preservatives, thermosonication, combined treatment of TS and NP, and storage time on Total phenolic content and Antioxidant activity of Sugarcane juices.

Phytochemical Analysis	Treatments	Storage Days				
		0	7	14	21	28
Total phenolic content (GAE mg/mL)	T0	5.11 ± 0.01^cd^	4.95 ± 0.02^ef^	4.83 ± 0.04^gh^	4.60 ± 0.01^hi^	4.40 ± 0.03^i^
	T1	5.56 ± 0.01^ab^	5.40 ± 0.02^ab^	5.19 ± 0.01^bc^	4.91 ± 0.03^fg^	4.52 ± 0.03^hi^
	T2	5.42 ± 0.02^jk^	5.30 ± 0.02^fg^	5.21 ± 0.02^de^	5.03 ± 0.02^c^	4.84 ± 0.01^a^
	T3	5.65 ± 0.01^a^	5.61 ± 0.02^a^	5.56 ± 0.01^ab^	5.47 ± 0.03^ab^	5.36 ± 0.02^b^
Antioxidant Activity (%)	T0	67.26 ± 0.03^ef^	62.13 ± 0.01^gh^	60.91 ± 0.01^hi^	57.74 ± 0.02^ij^	54.51 ± 0.04^j^
	T1	78.09 ± 0.01^b^	77.90 ± 0.02^b^	75.46 ± 0.03^bc^	73.31 ± 0.01^bc^	70.47 ± 0.01^cd^
	T2	68.09 ± 0.02^de^	68.02 ± 0.03^de^	67.40 ± 0.01^ef^	65.36 ± 0.02^ef^	63.48 ± 0.02^fg^
	T3	87.05 ± 0.03^a^	86.52 ± 0.01^a^	85.69 ± 0.02^a^	83.02 ± 0.02^a^	80.06 ± 0.02^ab^

Different letters in a column denote significant results (p ≤ 0.05), whereas the same letters denote non-significant results. TS = Thermosonication; NP = Natural Preservatives.

T0 = Control sample without natural preservatives and thermosonication.

T1 = Non thermosonicated Sugar cane juice with natural preservatives.

T2 = Thermosonocated sugar cane juice with no natural preservatives.

T3 = Thermosonicated Sugar cane juice with natural preservatives.

#### 3.3.1 Total phenolic content

The T1 after 2 weeks and the T0 samples during storage exhibited a significant decrease in total phenolic content (TPC) as shown in Table 6, likely due to microbial degradation and enzymatic activity. Similar observations were reported in pineapple juice by (Mala et al., 2021), suggesting that the decline in phenolic compounds correlates with deteriorating juice quality, leading to browning and sediment formation. Furthermore, oxidative degradation and polymerization with proteins and other chemical reactions occur during storage, contributing to the decrease in total phenolic content. (Khan et al., 2021).

T1 samples, treated with NP such as lemon and ginger, exhibited an increase in TPC (Table 6), also in agreement with Bag et al. (2022). This increase may be attributed to the bioactive phenols present in ginger. (H. Wang et al., 2022). Research by Khan et al. (2021). The enhancement of TPC, total flavonoids, and antioxidant value in SCJ treated with NP compared to the control samples was highlighted. T2 samples, subjected to TS treatment, showed a significant improvement in total phenolic content, consistent with findings reported by Yıkmış et al. (2025) and Nayak et al. (2020). This may be due to the extraction ability of TS treatment; thus, it has been observed positive effect of TS on TPC has been observed, as in these processes, there are releases of phenolic acids in bond form due to the cavitation effect. (Abid et al., 2014). TS treatments effectively retained the phenolic content of SCJ during storage (Table 6), likely due to the inactivation of enzymes during the TS process.

T3 samples demonstrated a significant increase in TPC compared to T2, T1, and T0, possibly due to the combined effects of NP and TS, as thermosonication treatment denatures the cell wall and causes the release of phenolic compounds from the juice sample. Additionally, T3, followed by T2, has the maximum retained total phenolic content during storage, as shown in Table 6. The retention of phenolic content during storage in T3 samples may be attributed to the inactivation of enzymes and microbial destruction achieved by TS treatment at lower temperatures and shorter durations. (Abid et al., 2014; Silva et al., 2025). This study contradicts (Zhao et al., 2021) Study outcomes in the case of total phenolic content.

#### 3.3.2 Antioxidant activity

Antioxidants (AA) play a crucial role in preventing or delaying the oxidation of substrates (living cells). The DPPH method was employed to evaluate the free radical scavenging activity of the control and treated SCJ. In T1, treatment samples displayed higher AA compared to the T2 sample, attributed to the presence of ascorbic acid in lemon and the high antioxidant potential in ginger. Activity also significantly increases in T2 samples after TS treatment and remains more stable during storage than in T1 and T0 samples, as shown in Table 6, and this effect was also noted in their study. (Nayak et al., 2020; Yıkmış et al., 2025). The reasons behind the increase of AA in TS-treated SC juice may be linked with increase in the extraction of total polyphenols, carotenoids, and other antioxidants due to the mechanical effect of the cavitation process and implosion of bubbles. (Fatima et al., 2023).

After treatment in the T3 sample AA was significantly higher compared to other samples, indicating a synergistic effect between TS and NP as shown in Table 6. Natural antimicrobials (ginger and lemon) which are a good source of antioxidants and TS have a good extraction ability which causes the increments of antioxidant levels in SCJ. This effect has also been noted in their study. (Zhao et al., 2021) has utilized the TS treatment combined with niacin preservative, and (Casco et al., 2022) Has utilized US treatment with natural antimicrobials (green tea extract), increased the TPC and antioxidant capacity, enhancing product stability during storage. And during storage, control samples showed a significant decrease in AA This may be due to enzymatic and oxidation processes, consistent with findings by (Mala et al., 2021). While T3 followed by T2 samples maintained high stability due to maximum denaturation PPO activity, which decreased the enzymatic browning, this effect of increasing AA and its stability during storage after combined treatment of the two preserving techniques has been noted by Silva et al. (2025) and Fatima et al. (2023).

### 3.4 Microbial analysis

Juice (SCJ) is prone to rapid spoilage due to its high moisture content, nutrient richness, and sugar content, providing an optimal environment for spoilage microorganisms.(Adulvitayakorn et al., 2020). Microbiological assays for determining shelf life commonly rely on total plate count, yeast, and mold counts, with maximum acceptable levels set at 4 and 3 log CFU/mL, respectively. (Geremias-Andrade et al., 2020).

Microbial load serves as an indicator of safety and shelf life for SCJ. TPC and YMC increase during storage (Table 7), albeit at a lesser rate in treated samples stored at refrigerated temperatures, as in the study of Pradhan et al. (2020). The TPC and YMC in SCJ in this study were lower than in other studies, as noted in their study. (Adulvitayakorn et al., 2020). This may be due to the use of peeled sugarcane rinds for juice extraction, this effect has also been noted by (Younos & Embaby, 2024). Peeled sugarcane rind, having had mud, black spots, and other contaminants removed, contains less TPC and YMC compared to unpeeled sugarcane rind. Also.

**Table 7:** Effect of natural preservatives, thermosonication, combined treatment of TS and NP, and storage time on the Microbial Analysis of Sugarcane juices.

Microbial Analysis	Treatments	Storage Days				
		0	7	14	21	28
Total plate count (log CFU/mL)	T0	5.41 ± 0.1^b^	5.56 ± 0.1^cd^	5.73 ± 0.2^c^	5.91 ± 0.3^b^	6.17 ± 0.4^a^
	T1	3.30 ± 0.3^g^	3.45 ± 0.2^g^	3.68 ± 0.3^f^	3.82 ± 0.1^f^	4.18 ± 0.2^e^
	T2	1.65 ± 0.1^ij^	1.71 ± 0.3^ij^	1.77 ± 0.1^i^	1.85 ± 0.2^h^	1.97 ± 0.3^h^
	T3	1.37 ± 0.2^m^	1.41 ± 0.1^lm^	1.47 ± 0.1^kl^	1.55 ± 0.4^jk^	1.65 ± 0.3^jk^
Yeast and Mold count (log CFU/mL)	T0	4.35 ± 0.3^d^	4.46 ± 0.1^cd^	4.58 ± 0.2^bc^	4.71 ± 0.3^b^	4.87 ± 0.2^a^
	T1	2.21 ± 0.4^f^	2.28 ± 0.2^f^	2.34 ± 0.4^f^	2.49 ± 0.3^e^	2.56 ± 0.3^e^
	T2	1.39 ± 0.2^ij^	1.44 ± 0.2^hi^	1.48 ± 0.3^gh^	1.55 ± 0.4^gh^	1.63 ± 0.1^g^
	T3	1.02 ± 0.1^m^	1.05 ± 0.3^m^	1.11 ± 0.4^lm^	1.18 ± 0.2^jk^	1.24 ± 0.3^kl^

Different letters in a column denote significant results (p ≤ 0.05), whereas the same letters denote non-significant results. TS = Thermosonication; NP = Natural Preservatives.

T0 = Control sample without natural preservatives and thermosonication.

T1 = Non thermosonicated Sugar cane juice with natural preservatives.

T2 = Thermosonocated sugar cane juice with no natural preservatives.

T3 = Thermosonicated Sugar cane juice with natural preservatives.

#### 3.4.1 Total plate count

During storage, the control sample exhibited the highest TPC, as also observed in their study. (Khan et al., 2021). In T1, the addition of lemon juice, which lowers the pH, and ginger, which contains antimicrobial compounds, significantly reduced the TPC. (Beristain-Bauza et al., 2019; J. Wang et al., 2020). Preservatives, such as lemon and ginger, have been shown to decrease bacterial counts in SCJ to meet stringent microbiological safety guidelines. (Abid et al., 2014).

T2 samples treated with TS exhibited a significant reduction in TPC (Table 7), consistent with findings. (Adulvitayakorn et al., 2020). That used TS to decrease TPC. TS treatment at mild temperatures with sonication has been shown to reduce TPC, with reductions in bacterial and mold populations (disrupting cellular structures) observed even at non-detectable levels for a short time. Furthermore, TS treatment has been associated with a 5-log reduction in fruit juice processing, aligning with FDA recommendations. (Oladunjoye & Awani-Aguma, 2023).

T3 samples exhibited a highly significant reduction in TPC. Similar reductions in microbial populations have been reported in apple juice following combined treatments. (Zhao et al., 2021). Studies have demonstrated that combining treatment methods can enhance microbial inactivation and prevent contamination. For instance, the combination of TS and ε-polylysine treatment effectively reduced *B. cereus* activity and disrupted microbial metabolism, leading to suppressed microbial growth. (Y. Yang et al., 2022). Acidic conditions, particularly when combined with non-thermal processes (thermosonication), enhance the inactivation of vegetative bacteria by inducing stress-induced changes in cytoplasmic pH and membrane dysfunction. Incorporating naturally occurring antimicrobials into non-thermal processing techniques has proven to be an effective strategy for microbial control. (Balasubramanian & Balasubramaniam, 2010; Ross et al., 2003). Treated samples demonstrated slower growth during storage, notably T3, followed by T2 and then T1, as shown in Table 7.

#### 3.4.2 Yeast and mold counts

Yeast and mold counts (YMC) progressively increased during storage across all sugarcane juice (SCJ) samples, with the untreated control (T0) exhibiting the highest microbial loads by the end of the 28-day period (Table 7). In contrast, the T1 sample, treated with natural preservatives (NP) including lemon and ginger, demonstrated a notable reduction in YMC. This can be attributed to the antimicrobial and acidic properties of lemon, which is rich in citric acid, vitamin C, and flavonoids, as well as ginger, which contains bioactive compounds such as gingerol and shogaol. These constituents, along with the addition of salt, likely contributed to the inhibition of fungal growth and extended shelf life of SCJ (Beristain-Bauza et al., 2019; Younos & Embaby, 2024).

A more substantial reduction in YMC was observed in the T2 samples treated with thermosonication (TS), supporting earlier findings that ultrasound-induced cavitation—coupled with moderate heat—can effectively inactivate microorganisms (Adulvitayakorn et al., 2020). The mechanical disruption of microbial membranes, along with localized heating and free radical formation, are believed to contribute to the antifungal effects of TS (Oladunjoye & Awani-Aguma, 2023; Zhao et al., 2021).

Among all treatments, the combined approach (T3) yielded the **lowest YMC values** and the most stable microbial profile during storage. This suggests a synergistic effect between TS and NP, where ultrasound and thermal action enhance the diffusion and efficacy of natural antimicrobials while simultaneously reducing pH to levels less favorable for fungal growth. Previous studies have shown that thermosonication at 60 °C, pH 3.0, and water activity (aw) of 0.99 can effectively reduce fungal populations with lower thermal input (López-Malo et al., 2005).

Nevertheless, some residual mold and yeast growth was still detected in all samples, which may be attributed to the spore-forming nature of certain fungi, making them inherently more resistant to conventional and non-thermal preservation methods (Oladunjoye & Awani-Aguma, 2023). Therefore, while the combined TS and NP treatment significantly enhanced microbial safety and shelf life of SCJ, it may require further optimization or integration with additional hurdles to achieve complete microbial inactivation for commercial applications.

### 3.5 Sensory evaluation

Sensory analysis is crucial for evaluating consumer preferences and the acceptability of juices. The evaluation followed a precise scoring scale ranging from 9, representing excellence, down to 0, with the threshold for acceptability set at 6 (Radha Krishnan et al., 2014). The study evaluated all sensory attributes of SC juice under different treatments over 28 days at 4 °C. Statistical analysis revealed significant disparities (*p* < 0.05) in some sensory parameters (like color, appearance, and taste) between the treated SCJ variants and the control.

#### 3.5.1 Color

**Fig. 1:**
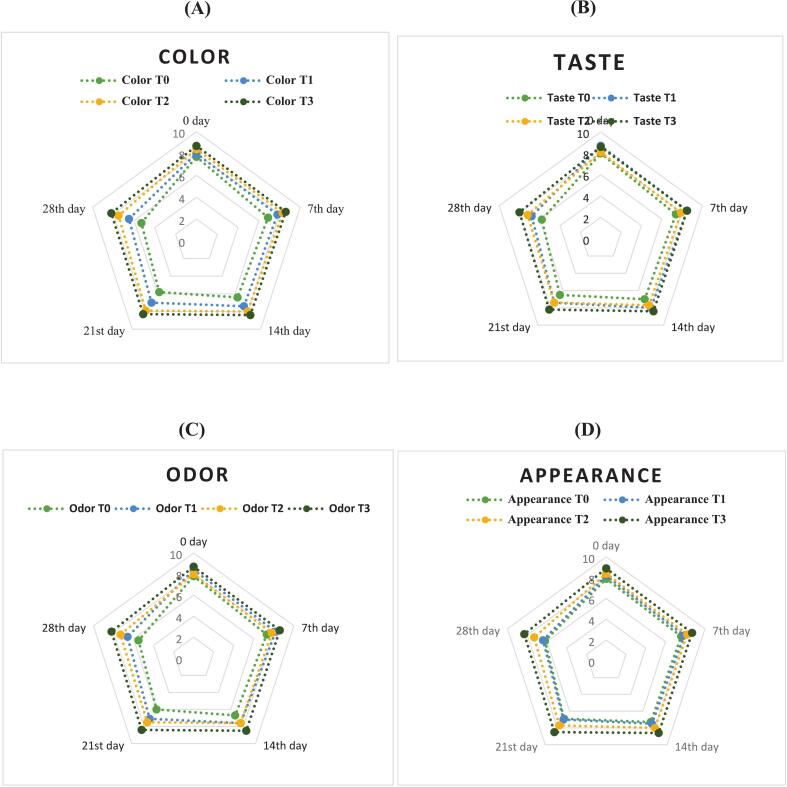

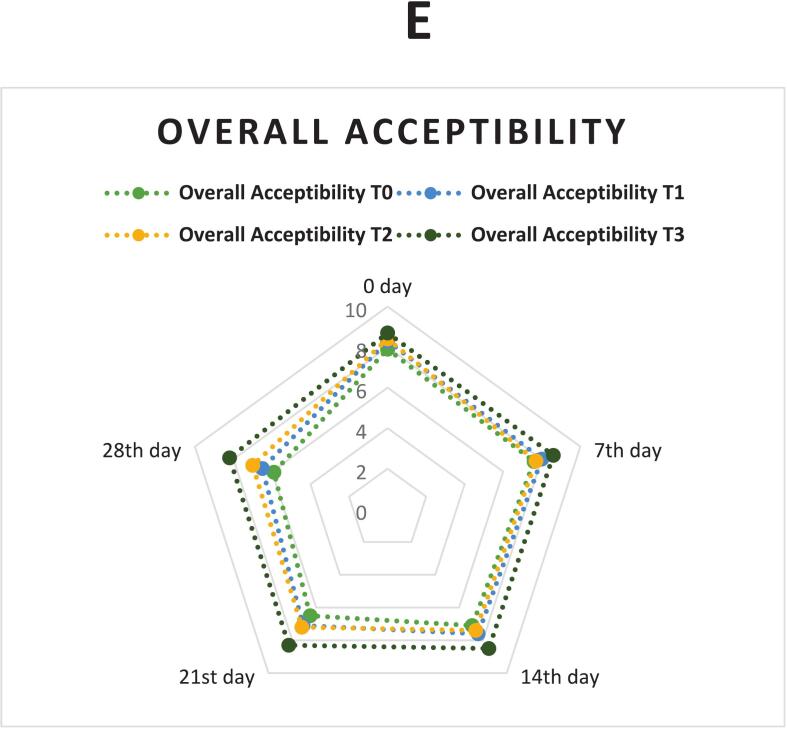
Sensory Analysis of T0, T1, T2 and T3 samples and treatment impact on the parameters **(A)** Color **(B)** Taste **(C)** Odor **(D)** Appearance, and **(E)** Overall acceptability score of all samples during storage. T0 = Control sample without natural preservatives and thermosonication. T1 = Non thermosonicated Sugar cane juice with natural preservatives. T2 = Thermosonocated sugar cane juice with no natural preservatives. T3 = Thermosonicated Sugar cane juice with natural preservatives.

The control sample (T0) showed significant color degradation during storage, as shown in Fig. 1.This indicates natural enzymatic browning and oxidation without any preservation. (Khan et al., 2021). Juice treated with NP in T1, including lemon and ginger, exhibited improvement in color and retained good color better than the control. While NP slowed down the degradation processes through its acidic and antioxidant properties, it was not sufficient alone for long-term preservation, as reported in their study. (Khare et al., 2012). TS treatment in T2 has significantly improved and maintained higher color stability, showing its effectiveness in inactivating enzymes and microorganisms responsible for color loss (Fig. 1) (Menelli et al., 2021).

The T3 sample of NP and TS showed the best results, with color scores remaining from Day 0 to the 28th. This improvement in color was due to the combined treatment, and these results are consistent with (Zhao et al., 2021). This synergy between NP's microbial inhibition and TS's enhancement of physical and chemical stability effectively preserved the juice's color, as shown in Fig. 1.

#### 3.5.2 Taste

Significant improvement in taste has been noted in T1 sample treated with NP and exhibited better taste retention than the control, over the period, as shown in Fig. 1, as also noted by Laelago Ersedo et al., 2023a, Laelago Ersedo et al., 2023b. The lemon and ginger provided antimicrobial and antioxidant benefits, slowing the degradation process and also have the potential to improve taste. However, the gradual decline during storage suggests that NP alone is not sufficient for long-term preservation. While the T0 sample showed a significant decline in taste, indicating natural degradation processes like microbial activity and enzymatic reactions. (Ali et al., 2023).

The TS treatment in T2 demonstrated a non-significant effect but maintained maximum taste scores after the T3 sample, as shown in Fig. 1 by Day 28, indicating its effectiveness in inactivating spoilage enzymes and microorganisms. (Oladunjoye & Awani-Aguma, 2023). In T3, the combined treatment of NP and TS proved the greatest improvement in taste scores. This minimal decline reflects the synergistic benefits, where NP provides microbial inhibition and TS enhances overall stability. This combined approach effectively preserved the taste of SC juice, showing significant taste stability during storage, as shown in Fig. 1.

#### 3.5.3 Odor

The T0 sample, without any treatment, exhibited a significant decline in odor quality, indicating natural spoilage processes and microbial activity. (Beristain-Bauza et al., 2019; Khare et al., 2012). In the T1 sample, after NP (lemon and ginger) addition, the odor also improved, but the odor quality remained for up to 2 weeks and then started decreasing, as shown in Fig. 1. This can be due to the use of only NP, which is not sufficient to preserve the odor effectively, like in T3.

No significant change occurred in the T2 sample and demonstrated more stable odor scores, as shown in Fig. 1. This method effectively reduced microbial load and inactivated spoilage enzymes, suggesting its viability for preserving odor quality. (Oladunjoye & Awani-Aguma, 2023). However, the combined treatment (T3) showed the best performance, as it has improved odor due to NP addition, as shown in Fig. 1. Their combined use offered superior preservation, ensuring minimal degradation over the 28-day storage period stored at 4 °C.

#### 3.5.4 Appearance

The sensory evaluation of SC juice appearance reveals significant differences among the three treatments. T1 exhibited improved stability in appearance compared to the control, and the appearance started decreasing after 2 weeks, as shown in Fig. 1. Ginger improves the appeal of the juice. (Laelago Ersedo et al., 2023a, Laelago Ersedo et al., 2023b). The antimicrobial and antioxidant properties of the preservatives contributed to slowing down the spoilage processes, though alone, they were not entirely effective in preventing degradation over the storage period. T2 demonstrated better results, with improved appearance, as has also been noticed in the (Oladunjoye & Awani-Aguma, 2023) Study. And during storage, T2 retained its appearance more than T1.

Significant improvement has been noted in the T3 sample, and it has shown the best performance in retaining appearance quality as compared to other samples; the best retaining effect of appearance during storage after combined treatment of the two techniques was also noted in their study in study. (Panigrahi et al., 2023). The appearance only slightly decreased during storage, as shown in Fig. 1. This minimal decline may be chemical reactions or remaining microbes. In conclusion, the combined approach (T3) effectively preserved the appearance of SC juice throughout the storage period, making it the most effective treatment; this effect was also noted in their study. (Ali et al., 2023).

#### 3.5.5 Overall acceptability

The T0 exhibited a pronounced decline in overall acceptability, with a decreasing effect during storage, indicating substantial quality degradation over time. (Zhao et al., 2021). T1, which involved the addition of NP, showed improved retention of overall acceptability compared to the control, as shown in Fig. 1. The natural preservatives provided antimicrobial and antioxidant benefits, which slowed the spoilage processes and helped maintain a better quality. (Laelago Ersedo et al., 2023a, Laelago Ersedo et al., 2023b). However, the decline was still significant, showing that NP alone was not entirely sufficient for long-term preservation. T2, utilizing TS, demonstrated better stability in overall acceptability than both T0 and T1as given in Fig. 1. TS effectively reduced microbial load and inactivated spoilage enzymes, thus preserving the juice quality more effectively.

However, T3, which combined NP and TS, performed best. The overall acceptability remained high, as shown in Fig. 1. In the study of (Zhao et al., 2021)Samples treated with the combined treatment of TS and natural antimicrobial agents maintained the highest overall acceptability during storage at 4 °C, with notable improvements in color. The combined treatment is particularly effective for extending the shelf life and maintaining the sensory appeal of SC juice stored at 4 °C, making it the most effective strategy among the evaluated methods.

## 4 Conclusion

In this study, the combined effect of thermosonication and natural preservatives on the quality of sugarcane juice (SCJ) was analyzed, revealing that this combined treatment significantly enhances microbial safety, enzyme inactivation, and overall juice quality during a 28-day storage period at 4 °C compared to using natural preservatives or thermosonication alone. The method effectively retained the total phenolic content and DPPH % levels, avoiding the drawbacks of conventional preservation methods. The combined treatment of thermosonication and natural preservatives likely stabilizes the juice by preventing particle aggregation and sedimentation, thereby maintaining higher turbidity and viscosity. Additionally, factors such as pH stabilization, antimicrobial effects, and inhibition of enzymatic activity contribute to preserving the juice's rheological and physical properties and extending its shelf life. This study highlights the potential of combining thermosonication and natural preservatives as a novel preservation strategy for sugarcane juice, offering substantial improvements in quality, safety, and shelf life. Beyond its laboratory-scale effectiveness, this method holds strong commercial potential for adoption by small- and medium-scale juice processors, especially in sugarcane-rich regions. Given its low environmental impact and minimal reliance on synthetic additives, the technique aligns with modern clean-label trends. Furthermore, the approach is amenable to scaling and integration into existing production lines with limited additional investment. Future research may explore continuous-flow TS systems and pilot-scale trials to further support industrial upscaling and commercialization.

## CRediT authorship contribution statement

**Tehmina Bibi:** Writing – review & editing, Writing – original draft, Visualization, Software, Resources, Methodology, Formal analysis, Data curation. **Atif Liaqat:** Writing – review & editing, Writing – original draft, Visualization, Supervision, Software, Formal analysis, Data curation, Conceptualization. **Tariq Mehmood:** Writing – review & editing, Writing – original draft, Software, Methodology, Formal analysis, Data curation, Conceptualization. **Rabia Iqbal:** Writing – review & editing, Visualization, Resources, Project administration, Formal analysis. **Muhammad Nadeem:** Writing – review & editing, Software, Resources, Investigation, Data curation, Conceptualization. **Ashiq Hussain:** Writing – review & editing, Validation, Software, Methodology, Formal analysis, Data curation. **Farhan Saeed:** Writing – review & editing, Validation, Software, Methodology, Investigation. **Muhammad Afzaal:** Writing – review & editing, Validation, Software, Methodology, Investigation, Conceptualization. **Suleiman A. Athawab:** Writing – review & editing, Visualization, Resources, Methodology, Investigation, Funding acquisition, Conceptualization. **Robert Mugabi:** Writing – review & editing, Software, Project administration, Formal analysis, Data curation. **Yash D. Jagdale:** Writing – review & editing, Validation, Software, Investigation, Conceptualization. **Gulzar Ahmad Nayik:** Writing – review & editing, Supervision, Project administration, Investigation, Formal analysis. **Tawfiq Alsulami:** Writing – review & editing, Software, Methodology, Funding acquisition, Formal analysis.

## Funding

The authors extend their appreciation to Ongoing Research Funding Program, (ORF-2025-641), King Saud University, Riyadh, Saudi Arabia.

## Declaration of competing interest

The authors declare that they have no known competing financial interests or personal relationships that could have appeared to influence the work reported in this paper.

## Data Availability

Data will be made available on request.
